# Climate Change Risks and Conservation Implications for a Threatened Small-Range Mammal Species

**DOI:** 10.1371/journal.pone.0010360

**Published:** 2010-04-29

**Authors:** Naia Morueta-Holme, Camilla Fløjgaard, Jens-Christian Svenning

**Affiliations:** 1 Ecoinformatics and Biodiversity Group, Department of Biological Sciences, Aarhus University, Aarhus, Denmark; 2 Department of Wildlife Ecology and Biodiversity, National Environmental Research Institute, Aarhus University, Rønde, Denmark; University of Durham, United Kingdom

## Abstract

**Background:**

Climate change is already affecting the distributions of many species and may lead to numerous extinctions over the next century. Small-range species are likely to be a special concern, but the extent to which they are sensitive to climate is currently unclear. Species distribution modeling, if carefully implemented, can be used to assess climate sensitivity and potential climate change impacts, even for rare and cryptic species.

**Methodology/Principal Findings:**

We used species distribution modeling to assess the climate sensitivity, climate change risks and conservation implications for a threatened small-range mammal species, the Iberian desman (*Galemys pyrenaicus*), which is a phylogenetically isolated insectivore endemic to south-western Europe. Atlas data on the distribution of *G. pyrenaicus* was linked to data on climate, topography and human impact using two species distribution modeling algorithms to test hypotheses on the factors that determine the range for this species. Predictive models were developed and projected onto climate scenarios for 2070–2099 to assess climate change risks and conservation possibilities. Mean summer temperature and water balance appeared to be the main factors influencing the distribution of *G. pyrenaicus*. Climate change was predicted to result in significant reductions of the species' range. However, the severity of these reductions was highly dependent on which predictor was the most important limiting factor. Notably, if mean summer temperature is the main range determinant, *G. pyrenaicus* is at risk of near total extinction in Spain under the most severe climate change scenario. The range projections for Europe indicate that assisted migration may be a possible long-term conservation strategy for *G. pyrenaicus* in the face of global warming.

**Conclusions/Significance:**

Climate change clearly poses a severe threat to this illustrative endemic species. Our findings confirm that endemic species can be highly vulnerable to a warming climate and highlight the fact that assisted migration has potential as a conservation strategy for species threatened by climate change.

## Introduction

Global temperature is expected to rise at a rapid rate during the 21^st^ century [1]. Anthropogenic climate change is already affecting the physiology, phenology, behaviour and distribution of many species [2]–[8] and these impacts can be expected to intensify. Past climate change has caused radical biological changes involving dramatic range shifts as well as extinctions [5], [9]–[11]. It is increasingly clear that imminent climate changes will strongly affect biodiversity and ecosystems [5], [12] and may potentially result in high extinction rates around the world (e.g., [13]–[17]).

The large proportion of species with narrow ranges (hereafter, endemic species) are a special concern: their small range is a liability *per se* [18] and they are likely to be more dispersal-limited than other species and, therefore, less able to track a rapidly shifting climate [19], [20]. However, the extent to which current climate limits the distribution of endemic species is unclear; notably, richness of endemic species often correlates poorly with current climate and is more strongly related to factors describing long-term survival and speciation (e.g., [21], [22]). Nevertheless, a recent study found areas with high numbers of small-range species to be colder and located at higher elevations than surrounding regions, suggesting that these are interglacial relict areas for cold-adapted species with a high vulnerability to future global warming [23].

Given the high extinction risk faced by species unable to adapt or disperse at a rate that is sufficient to track anthropogenic climate change, assisted migration has been suggested as a possible conservation strategy [24], [25]. This would involve translocating species to currently unoccupied, but environmentally suitable areas that are likely to remain suitable over the next 100 years or more, in cases where other conservation strategies are unlikely to be sufficient to ensure their survival [24], [25]. There are many examples where biological introductions have had negative biological and socioeconomic effects, and great care is therefore needed before implementing assisted migration [24]. Accordingly, Hoegh-Guldberg et al. [24] outline a decision framework for assessing potential species translocations according to the need for this conservation action, its technical feasibility, and the biological and socioeconomic costs-benefits. An important first step in the framework consists of assessing to what extent more conventional approaches (e.g., reducing local stressors, reducing habitat fragmentation, or *ex situ* conservation) would suffice to protect a species in the face of climate change.

Here, we provide a detailed assessment of the climate sensitivity and potential distributional impacts of 21^st^ century climate change for an illustrative endemic species limited to a restricted part of the Mediterranean region. This region is rich in endemic species and is expected to experience particularly severe global-change-driven biodiversity losses over the 21^st^ century [5], [12], [15]. The study species is the Iberian desman *Galemys pyrenaicus* (E. Geoffroy Saint Hilaire, 1811), a small semi-aquatic mammal endemic to the Iberian Peninsula. It is considered “Vulnerable” in the 2007 IUCN Red List of Threatened Species and it is listed in Annexes II and IV of the European Habitats Directive (92/43/ECC) and Appendix II of the Bern Convention. It belongs to the subfamily Desmaninae (Soricomorpha: Talpidae), which has only one other extant species: the Russian desman *Desmana moschata*, which occurs in Russia, Ukraine and Kazakhstan [26], [27]. The present distribution of *G. pyrenaicus* covers the Pyrenees and northern Iberian Peninsula, where it is found in cold, highly oxygenated mountain rivers and streams, feeding almost exclusively on aquatic invertebrates [26], [28], [29]. Given its preference for cool habitats, *G. pyrenaicus* is likely to be particularly vulnerable to global warming (cf. [23]), similar to certain other cool-adapted montane mammal species (e.g., [13]). *Desmana moschata* was widely distributed in Europe during the last Ice Age and contracted to its current limited range during the subsequent warming [30]–[32]. However, it is unclear to what extent *G. pyrenaicus* is directly sensitive to warm temperatures; other climatic factors that may limit its distribution are high variability in annual water discharge rate and low precipitation [33], [34]. In addition, climate will clearly not be the only determinant of *G. pyrenaicus*' range dynamics over the 21^st^ century. During the last several decades, the distribution of *G. pyrenaicus* has contracted; this is probably driven mainly by habitat loss and fragmentation due to the destruction of riversides and the construction of hydroelectric power stations and river contamination, the latter creating dispersal barriers between non-polluted rivers [35]–[37].

In the present study, we used species distribution modeling to examine range determinants, climate change sensitivity, potential global warming impacts, and conservation implications for *G. pyrenaicus.* Species distribution modeling is widely used as a tool in ecology and conservation biology [38], [39] and is one of the main feasible approaches to get a comprehensive, quantitative understanding of the potential complexity of factors limiting the range of rare, cryptic species such as *G. pyrenaicus*. Nevertheless, it is important to be aware of potential problems associated with this approach, especially concerning the selection of explanatory variables, e.g., the risk of under-representing potentially important non-climatic variables, spatial autocorrelation, and scale issues [cf. 40, 41]. We directly addressed these issues in our study by including a carefully selected set of ecologically motivated climatic and non-climatic range predictors, emphasizing variables for which there were *a priori* reasons to think they may be important, and maximizing the geographic independence of the training and test data sets. Furthermore, we analyzed the distribution of *G. pyrenaicus* at a relatively fine spatial resolution (10 km) and for its main area of occurrence (Spain); a climatically diverse region. As a result, we were confident that we were estimating the climate sensitivity of *G. pyrenaicus*, while largely disregarding the broad-scale historical range constraints that are likely to dominate the distribution of endemic species within broader regions [20], [42]. We addressed the following specific questions:

1. How important is current climate relative to other factors in controlling *G. pyrenaicus*' distribution at a 10-km scale in Spain? Which specific climatic factors are the most important?
2. To what extent will *G. pyrenaicus* be threatened by global warming?
3. What is the scope for assisted migration [24] as a conservation strategy for *G. pyrenaicus* in a warming climate?

## Methods

### Study region and distribution data

The main study region was continental Spain (493,518 km^2^), which is a climatically diverse region with a longitudinal gradient in precipitation and a latitudinal gradient in both temperature and precipitation. However, we also used data from across all of Europe (c. 34°−71°N, 32°E −11°W) to assess European-scale conservation possibilities for *G. pyrenaicus* under future global warming.

**Figure 1:**
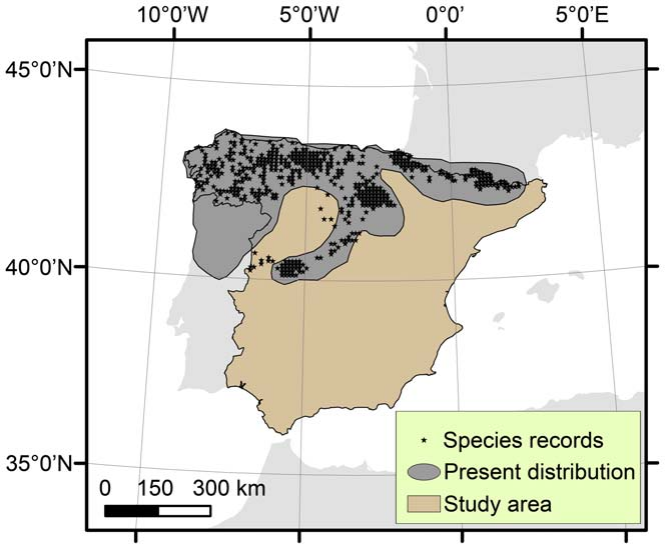
Distribution of *Galemys pyrenaicus*. The present distribution of *Galemys pyrenaicus*, according to IUCN (grey shading) [27], and its occurrence in Spain, according to the Spanish atlas on terrestrial mammals (stars) [29].

Distributional data for *G. pyrenaicus* were available from the Spanish atlas of terrestrial mammals [29]. The species was present in 328 out of 5115 10 km×10 km UTM (Universal Transverse Mercator) grid cells (Fig. 1). The aquatic and nocturnal habits of *G. pyrenaicus* make it difficult to detect [43], so we considered the distributional data as presence-only data [44].

### Environmental data

We initially considered a total of 20 variables (Table 1) representing the main factors that are considered important range determinants for *G. pyrenaicus*: topography, climate and human impact. The topographic and climatic variables were specifically selected because the occurrence of *G. pyrenaicus* has been reported to be associated with mountainous areas, cold and highly oxygenated rivers and streams, low variability in annual water discharge rate and high precipitation (see Introduction). The climate and topography variables were extracted from the WorldClim data base at 30″ (∼1-km) resolution for the period 1950–2000 (http://www.worldclim.org/; [45]). Human impact was represented by two variables: the human population density in the year 2000 [46] and the human footprint, an estimate of human influence based on population density, land transformation, accessibility and infrastructure data from the 1960s to 2001 [47]. We converted all predictor variables to their means (except for altitude, which was converted to its standard deviation and range) for each 10 km×10 km grid cell.

**Table 1:** The initial set of environmental variables and their range of values across all 10 km×10 km grid cells in continental Spain.

Variables	Code	Values
Altitude range^a^ (m)	ALT_RANGE	0–2080
**Altitude standard deviation** b **(m)**	**ALT_STD**	**0**–**509.30**
Annual mean temperature^c^ (°C)	AMT	0.25–18.50
Monthly minimum temperature^d^ (°C)	MMT	−6.28–12.72
**Mean summer temperature** e **(°C)**	**MST**	**7.13**–**26.77**
**Mean winter temperature** f **(°C)**	**MWT**	**−5.69–13.09**
Maximum summer temperature^g^ (°C)	MXST	8.36–28.23
Annual precipitation^h^ (mm)	PANN	221.66–1520.23
Minimum precipitation^i^ (mm)	PMIN	0–98
Precipitation seasonality^j^ (mm)	PSEA	8.22–63.56
Summer precipitation^e^ (mm)	PSUM	3.33–117.00
Winter precipitation^f^ (mm)	PWIN	0–362
**Water balance** k **(mm)**	**WBAL**	**−814.84**–**1341.68**
Absolute minimum temperature^l^ (°C)	TMIN	−3.05–−0.49
Annual temperature range^m^ (°C)	TR	8.8–20.29
Temperature seasonality^j^ (°C)	TS	3.16–6.97
Water balance seasonality^j^ (mm)	WB_SEA	18.71–83.09
**Summer water balance** e **(mm)**	**WB_SUM**	**−123.22**–**80.98**
Human population density in year 2000^n^ (persons pr km^2^)	HPD00	0.01–13463.00
**Human footprint** o	**HFOOTP**	**0.00**–**79.01**

The variables used in the distribution modeling for *Galemys pyrenaicus* are bold-faced.

a Difference between maximum and minimum altitude.

b Standard deviation of values.

c Average of monthly mean daily temperatures.

d Monthly mean temperature of the coldest month.

e Mean for June, July and August.

f Mean for December, January and February.

g Maximum for June, July and August.

h Sum of monthly mean precipitation over the year.

i Minimum monthly value.

j Standard deviation of mean monthly values.

k Yearly sum of the monthly differences between precipitation and potential evapotranspiration, following [68].

l Following [77].

m Difference between maximum and minimum monthly value.

n [46].

o [47].

Using many correlated predictors in species distribution modeling may result in over-parameterization and loss of predictive power [13] as well as lessening interpretability. For predictor pairs with Pearson r ≥0.9, we only retained one of the variables for the modeling [48] by selecting the variable with the strongest biological interpretability and the smallest correlation to the other predictor variables (Tables 1, 2). The exceptions to this were mean summer temperature (MST) and summer water balance (WB_SUM; Table 2), which were both retained, as they could be important for *G. pyrenaicus'* distribution through different mechanisms (see Discussion). The final set of predictors represented topography (altitude standard deviation, ALT_STD), temperature (MST; mean winter temperature, MWT), seasonal and overall climatic water balance (WB_SUM; annual water balance, WBAL) and human impact (human footprint, HFOOTP; Table 1).

**Table 2:** Pearson's correlations between the variables used in the distribution modeling for *Galemys pyrenaicus*.

	ALT_STD	HFOOTP	HPD00	MST	MWT	WB_SUM
**HFOOTP**	−0.211					
**HPD00**	−0.071	0.319				
**MST**	−0.491	0.194	0.055			
**MWT**	−0.365	0.370	0.192	0.748		
**WB_SUM**	0.505	−0.151	−0.014	**−0.951**	−0.679	
**WBAL**	0.537	−0.218	−0.024	−0.876	−0.549	0.883

Altitude standard deviation (ALT_STD), human footprint (HFOOTP), human population density (HPD00), mean summer temperature (MST), mean winter temperature (MWT), summer water balance (WB_SUM) and annual water balance (WBAL). Bold-face indicates |r|>0.9.

We based model projections into the future on predicted average climate data for the period 2070–2099 for the four Intergovernmental Panel on Climate Change climate change scenarios (A1 (A1FI), A2, B1 and B2) [49], which represent different assumptions regarding economic growth, technology, demographic changes and governance [1]. Warming is in all cases expected to be the greatest in south-western Europe, with summer temperature increases sometimes exceeding 6.0°C above summer temperature average for the years 1961–1990 in parts of France and the Iberian Peninsula, while precipitation is expected to decrease, especially during summer [4].

### Distribution modeling

The main modeling method used was MAXENT, a machine-learning method that estimates a species' distribution across a study area by calculating the probability distribution of maximum entropy subject to the constraint that the expected value of each feature under this estimated distribution should match its empirical average [50]. The MAXENT method is among the best-performing modeling approaches for presence-only occurrence data [50], [51]. We implemented MAXENT using version 3.2.1 (http://www.cs.princeton.edu/~schapire/maxent/). We used default values for the convergence threshold (10^−5^), maximum number of iterations (500) and the newly introduced logistic output format [52]. The logistic output can be interpreted as an estimate of the probability of presence (ranging from 0–1), conditioned on the environmental variables in each grid cell [52].

To assess the factors determining the distribution of *G. pyrenaicus* and to develop predictive distribution models, we fitted and evaluated the models including all predictor variables (with one exception: the highly correlated MST and WB_SUM were not included in the same model) and we progressively developed simpler models by removing the variables that contributed the least predictive power (lowest test gain according to the jackknife evaluation, see below; Table 3). Araújo and New [53] recommended using ensemble forecasting in order to obtain more robust predictions. We therefore also performed an ensemble prediction, namely the agreement regarding the predicted distribution between the five final models.

**Table 3:** The seven MAXENT distribution models for *Galemys pyrenaicus*.

Model	ALT STD	HFOOTP	MST	MWT	WBAL	WB SUM	AUC			Presence threshold
							Random	West	East	
1	X	X		X	X	X	**0.876**	0.737	**0.781**	-
2	X	X	X	X	X		**0.880**	**0.802**	**0.828**	0.353
3	X	X		X		X	**0.860**	0.725	0.730	-
4			X		X		**0.871**	**0.824**	**0.867**	0.323
5	X		X		X		**0.875**	**0.820**	**0.851**	0.318
6					X		**0.861**	**0.918**	**0.860**	0.329
7			X				**0.863**	**0.837**	**0.864**	0.369

Environmental predictor variables, model performance according to the test–AUC and presence threshold chosen for each model are given. The model performance was computed on different test data sets: 30% of *G. pyrenaicus* presence data drawn at random (Random), or selected as the 30% most westerly (West) or easterly (East) presence cells. AUC-values >0.75 (good predictive ability) are bold-faced. Presence thresholds were set at the 10^th^ percentile training presence.

Predictions from different modeling approaches can vary substantially (e.g., [54]). To ensure that our results were not dependent on the specific modeling algorithm used, we performed supplementary analyses using an alternative and, in terms of climate sensitivity, more conservative modeling approach, BIOCLIM [55]. In contrast to MAXENT, BIOCLIM is a profile method that does not utilize pseudo-absence (background) data [51] and the two methods have performed quite differently in recent modeling comparisons [51], [56]. We parameterized the BIOCLIM models using the minimum and maximum, 2.5^th^ and 97.5^th^ percentiles and 10^th^ and 90^th^ percentiles of the observed environmental values within the species' current distribution range in the study area. Suitable areas for the species were predicted when all of the environmental variables fell in the inner range of these limit values, thus defining four levels of suitability varying from unsuitable (outside the observed range) to highly suitable (inside the conservative 10–90 percentile interval). In the BIOCLIM modeling, only the predictor combinations of the five best MAXENT models were used (see Results).

ArcGIS 9.2 (ESRI, Redlands, CA) was used for all GIS operations and the BIOCLIM modeling.

### Model evaluation

To assess the predictive capacity of the MAXENT models, we split the data so that models were calibrated using 70% of the observed species data (training data) and evaluated for predictive accuracy using the remaining 30% of the data (test data). We measured the accuracy of the MAXENT models using the Area Under the receiver operating characteristic Curve (AUC) which is a threshold-independent measure of a model's ability to discriminate between absences and presences [57] and a standard method to assess the accuracy of predictive distribution models (e.g., [58]–[60]). An AUC value of 0.5 indicates that the model has no predictive ability, whereas a perfect discrimination between suitable and unsuitable cells will achieve the best possible AUC of 1.0. For presence-only occurrence data, AUC can be interpreted as the probability that the model assigns a higher score to a randomly chosen cell known to harbour the species than to a randomly chosen cell in which its presence is unknown [50]. Models with AUC >0.75 for both training and test data were accepted [51]. Spatial autocorrelation in species occurrences will cause a lack of independence between the test and training data sets if the division into training and test data is done randomly. This will cause an overoptimistic evaluation of model transferability, i.e., the predictive power of a model in new regions or time periods [38]. Although MAXENT has been shown to perform well in terms of transferability [61], we implemented a geographic partitioning to provide more independent training and test data and thereby provide more honest estimates of the models' predictive ability [38], [62]. The 70% most easterly presence cells were used as training data, while the remaining 30% were used as test data. We also did the converse partitioning, using the western 70% as the training data and the remainder as test data. In each case, all background data cells west or east of the partitioning longitude were also excluded. For comparison with previous studies, we also computed test AUCs based on random partitioning of the data into 70% training and 30% test data.

We used MAXENT's internal jackknife test to assess the importance of each environmental variable for predicting the distribution of *G. pyrenaicus* in Spain, rerunning a model with all six variables excluding each environmental variable in turn and also using each variable in isolation. The complete six-variable model was then compared to the jackknifed and single variable models. Comparison with jackknife tests on the five-variable models (where the correlated MST and WB_SUM were kept separated) showed no influence of the MST-WB_SUM correlation on the predictor rank order importance.

We derived presence-absence maps from the logistic suitability output from MAXENT using the 10^th^ percentile training presence threshold, which predicts absent the 10% most extreme presence observations, as these may represent recording errors, ephemeral populations, migrants, or the presence of unusual microclimatic conditions within a cell (e.g., [63]). After the application of this threshold, we compared the MAXENT and BIOCLIM models based on all the sample data to the realized distribution using Cohen's kappa statistic, which measures the proportion of correctly predicted sites correcting for the probability of agreement by chance [54].

### Model projection

To assess the impact of 21^st^ century climate change on *G. pyrenaicus,* we reran MAXENT models that performed well in the geographically partitioned tests with the complete sample data as training data and projected them onto the future climate scenarios for Spain. Conservatively, HFOOTP was kept constant at present levels in the future scenarios. The climate change impact was assessed by calculating the change in the suitable area for *G. pyrenaicus* based on the predicted presence-absence maps for the present-day and each of the four future climate change scenarios.

In order to evaluate the potential for implementing assisted migration as a conservation strategy for *G. pyrenaicus*, we identified suitable areas outside the present range of the species by projecting the two best MAXENT models across the whole of Europe, both under the present climate and the four 2070–2099 climate scenarios. As a conservative approach, we limited the projections to areas with an environment consistent with that currently occupied by *G. pyrenaicus*. Thus, we restricted them to mountainous regions by excluding areas with an altitude lower than 400 m, given that *G. pyrenaicus* populations very rarely occur below this altitude [33] and to regions with mean winter temperatures not lower than those found within the species' current distribution. The freezing of streams over longer periods could be a limiting factor, with similar effects on the access to food resources as drought. Additionally, very cold temperatures might have negative physiological impacts on *G. pyrenaicus*.

## Results

**Figure 2:**
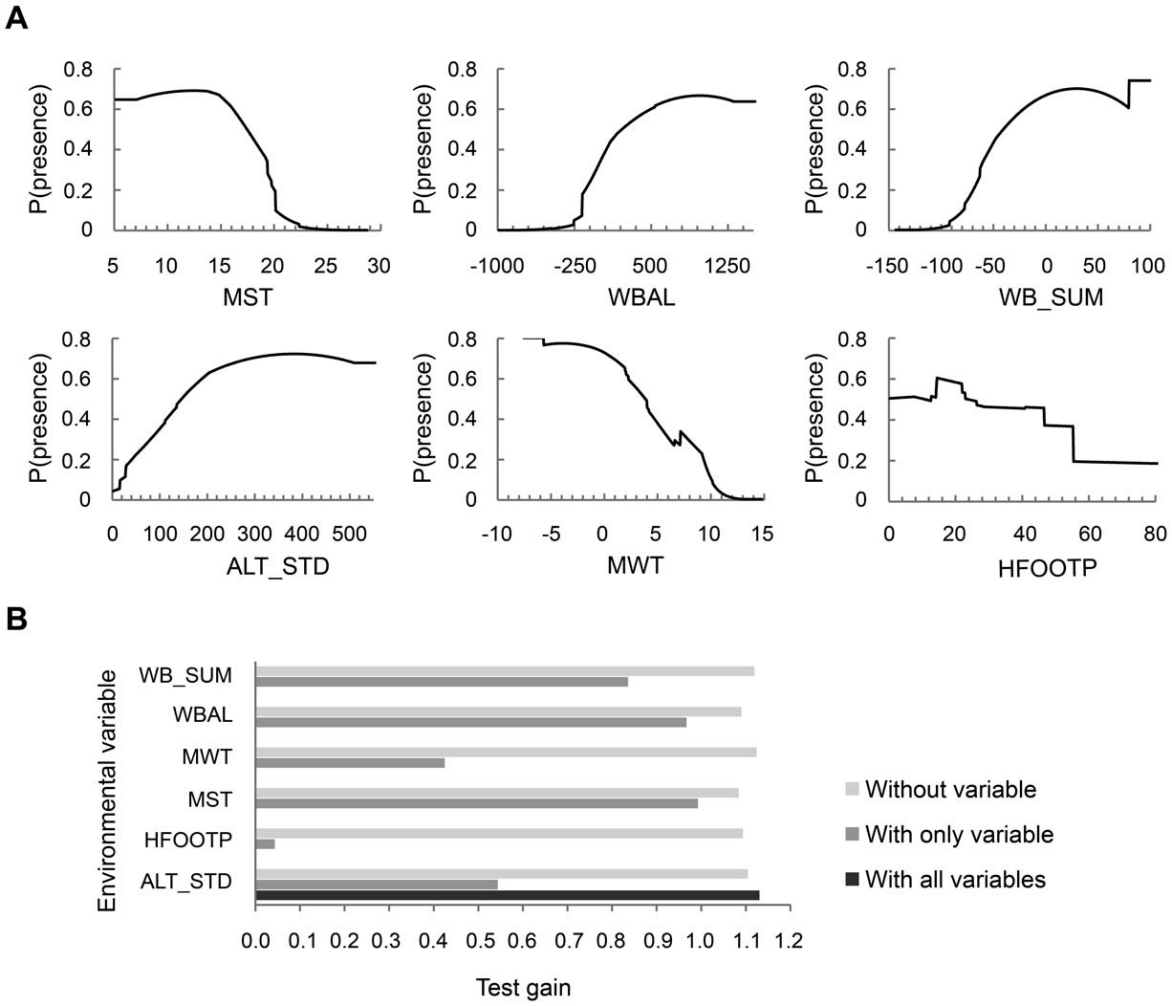
Results of the MAXENT model with all six explanatory variables selected for modeling. For acronyms, see Table 1. (**A**) Estimated response curves (logistic output: probability of presence). (**B**) Results of jackknife evaluation of the relative importance of the variables with respect to the test gain.

The probability that *G. pyrenaicus* was present was positively related to WBAL, WB_SUM and ALT_STD and negatively related to MST, MWT and HFOOTP (Fig. 2a). Hence, our results confirm that *G. pyrenaicus* occurs mainly where there is surplus precipitation, notably during the summer (i.e., consistent water flow), cool temperatures, steep terrain and little human impact. The jackknife evaluation procedure indicated that the climatic variables MST and WBAL were the strongest predictors and of equal strength, while HFOOTP was the weakest (Fig. 2b).

**Figure 3:**
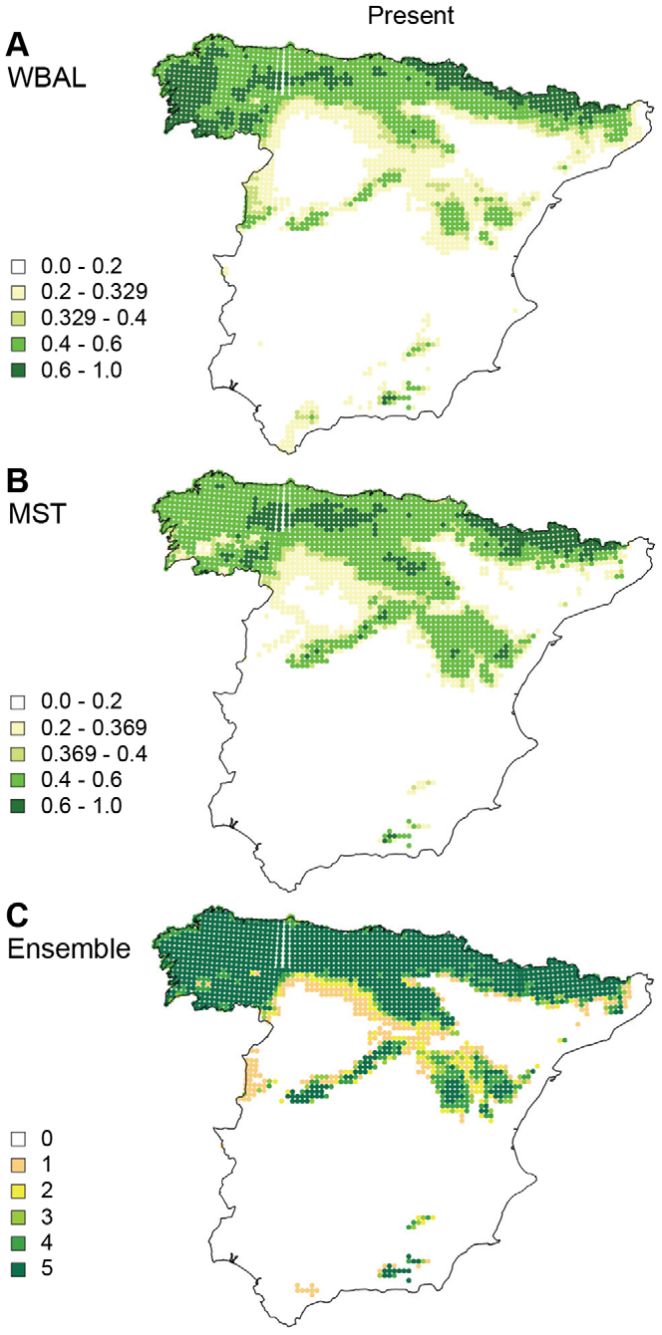
Present potential distribution of *Galemys pyrenaicus* in Spain. MAXENT predictions of the present potential distribution of *Galemys pyrenaicus* in Spain at a 10 km×10 km resolution: predictions based on (**A**) water balance (WBAL) and (**B**) mean summer temperature (MST). The predicted probability of presence, with values ranging from 0 to 1, is depicted by colours. The 10^th^ percentile training presence threshold is indicated (0.329 and 0.369, respectively). (**C**) Ensemble intersection: overlap of predicted presence among the five best models. The colours indicate the number of models predicting presence for each grid cell ranging from 0 to 5, based on the 10^th^ percentile training presence threshold (Table 3).

Comparing the seven MAXENT models, models 1 and 3 were rejected for use in the projections, as they both had test AUC values ≤0.75 (Table 3). The remaining five models that were selected for projections were based on one or several of the following variables: MST, WBAL, ALT_STD, MWT and HFOOTP. The five models produced concordant predictions (Fig. 3) and using solely MST or WBAL was sufficient to achieve good performance (Table 3, Fig. 3).

According to Cohen's kappa (Fig. S1) the MAXENT models performed better than the BIOCLIM models. Nevertheless, predictions from the BIOCLIM models were similar to those from the MAXENT models (Fig. S2), showing that our findings were relatively robust to the choice of modeling approach.

**Figure 4:**
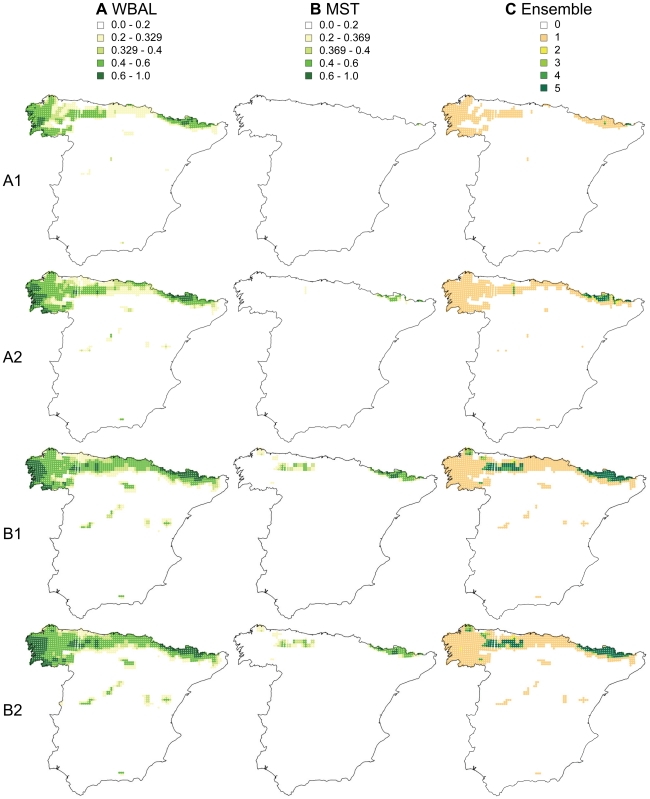
Future potential distribution of *Galemys pyrenaicus* in Spain. Projection of MAXENT distribution models for *Galemys pyrenaicus* in Spain onto four future climate scenarios for 2070–2099. (**A**) and (**B**) predicted probability of presence from projections of models based only on water balance (WBAL) or mean summer temperature (MST), respectively. The 10^th^ percentile training presence threshold is indicated (0.329 and 0.369, respectively). (**C**) Ensemble intersection: overlap of predicted presence among the five best models. Colours indicate the number of models predicting presence (based on the 10^th^ percentile training presence threshold) for each grid cell ranging from 0 to 5.

Projecting the selected five models onto the four climate change scenarios consistently predicted severe reductions by the period 2070–2099 in the environmentally suitable area for *G. pyrenaicus* in Spain (Table 4), with a strong northward range contraction (Fig. 4). The severity of the range reductions varied according to the climate scenario, with the A1 scenario causing 4 out of 5 models to predict near total loss of environmentally suitable conditions in Spain (Table 4, Fig. 4). The four models that included MST as a predictor consistently predicted the most dramatic declines (0.1–12% of the present potential distribution remaining), while losses were much more moderate, yet still dramatic (30–60% of the present potential distribution remaining), according to the WBAL model (Table 4, Fig. 4). This may be explained by the larger changes in MST predicted for 2100 relative to the predicted changes in WBAL: the average changes in the standardized values ranged 1.29 to 2.58 for MST, depending on the climate change scenario, but only −0.67 to −1.27 for WBAL.

**Table 4:** The predicted climate change impact on the distribution of *Galemys pyrenaicus* in Spain in 2070–2099 under four climate change scenarios.

Model		2	4	5	6	7	Ensemble-intersection
Predicted present area (km^2^)		127 500	155 100	149 700	149 300	167 900	113 700
**Change**	**A1**	0.3%	0.3%	0.3%	31.4%	0.1%	0.1%
	**A2**	3.8%	2.8%	4.0%	44.1%	1.4%	2.1%
	**B1**	12.4%	11.7%	12.4%	57.6%	7.0%	10.2%
	**B2**	12.2%	11.5%	12.4%	61.2%	6.7%	9.8%

The change in the predicted distribution (% of current predicted distribution) is shown for the five best MAXENT models. The ensemble intersection gives the predicted presence area and the changes herein that all five models agree upon.

**Figure 5:**
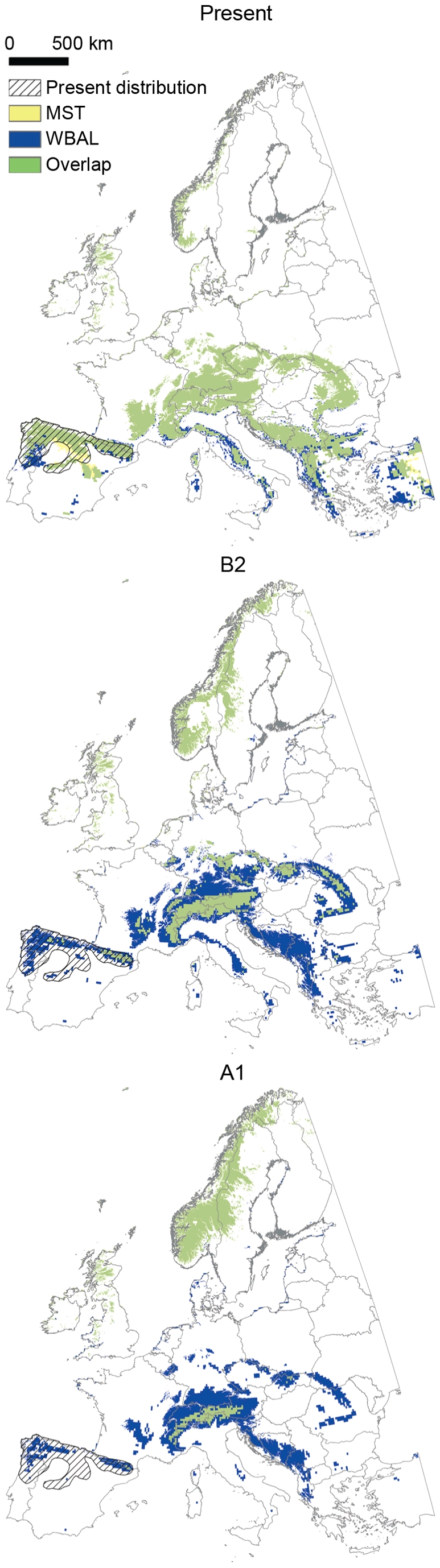
Present and future potential distribution of *Galemys pyrenaicus* in Europe. Suitable areas for *Galemys pyrenaicus* in Europe under the current climate and the B2 and A1 scenarios for 2070–2099, projected from MAXENT models based on water balance (WBAL) and mean summer temperature (MST). Areas with an altitude lower than 400 m and/or with a mean winter temperature lower than −5.687°C were conservatively set as unsuitable. *Galemys pyrenaicus*' present distribution is also shown [27].

Projecting the WBAL and MST models across Europe under current climate and the four climate scenarios showed major suitable areas beyond the current native range of *G. pyrenaicus*. In the period 2070–2099, large suitable areas were predicted to occur in Scotland and Scandinavia, even under the most severe (A1) scenario (Fig. 5). Other southern mountainous areas such as the Alps are also currently suitable, but do not harbour any *G. pyrenaicus* populations. As for Spain, the extent to which currently occupied areas will remain suitable by the end of this century depended on whether the distribution of *G. pyrenaicus* is controlled mostly by WBAL or MST (Fig. 5).

## Discussion

### Which factors determine the range of *G. pyrenaicus*?

The present distribution of the Iberian endemic mammal *G. pyrenaicus* was modeled as a function of climate, topography and human impact for the whole of Spain. The five best performing models according to the AUC values included combinations of three climate variables (MST, WBAL and MWT), topography (ALT_STD) and the human footprint (HFOOTP). The climatic variables WBAL and MST were each individually capable of predicting the current distribution of *G. pyrenaicus* accurately, providing evidence that climate clearly is the main current range determinant in Spain, at least among the variables considered and at the scale measured, despite local population declines caused by anthropogenic pressures, such as habitat destruction and pollution [35], [37], [43]. Importantly, our results confirm that the range of narrow endemics like *G. pyrenaicus* can be strongly related to climate [23].

Considering the relationships to individual environmental variables, our results agree well with the literature. The strong positive relationship with WBAL found in our study (Fig. 2a) is consistent with reports of higher occupancy rates in areas where the water discharge rate is high and regular [33], [34]. The dependence on a positive water balance is also obvious from the amphibious lifestyle of *G. pyrenaicus* and its dependence on benthic invertebrates as food [29]. The strong negative relationship to MST is also in agreement with the reported association of *G. pyrenaicus* with cold mountain streams [26], [36] and its biogeographic history, which has also been interpreted to indicate high temperatures as a limiting factor [37]. No studies have investigated the temperature sensitivity thresholds for this species or the mechanisms involved (direct physiological effects of heat stress, or indirect effects). Studies on other species have shown that mammals, despite being endothermic, can be highly sensitive to temperature. Notably, there is experimental evidence for heat stress intolerance in the ringtail possum (*Pseudochirops archeri*), a small montane mammal from Australia [13]. High mortality rates following periods of very high temperatures have also been reported for some species, e.g., Australian flying foxes [17]. Previous Quaternary warming events have been linked to population declines or range contractions for a number of mammal species, e.g., reindeer [11] and woolly mammoth [64]. In other cases, local extinctions have been explained by a combination of warming and drought as seen in the extinction of cool- and moist-adapted small mammal species in the North American Great Basin during the Middle Holocene [9]. It is not clear from our results to what extent WBAL and MST have independent effects. As there is a negative correlation between the two variables (Table 2), MST may largely be acting as a surrogate for WBAL, or vice versa. Nevertheless, considering the amphibious lifestyle of *G. pyrenaicus*, WBAL must clearly be important. A role for MST is also in line with the literature (see above), although it is noteworthy that *G. pyrenaicus'* only close relative *D. moschata* lives in a lowland region with relatively high summer temperatures (southern Russia, Ukraine and Kazakhstan).

The other environmental variables, MWT, HFOOTP and ALT_STD, had minor effects on the species' distribution at the scale studied. The literature points at human influence and topography as important limiting factors for this species [33], [36], [37]. Hence, the small effect of HFOOTP and ALT_STD on the predictive power of the models in the present study might be a consequence of the resolution of the study (10 km×10 km), which will not detect the influence of factors acting at smaller scales [65]. Furthermore, the geographic scope may also play a role. The previous ecological studies of *G. pyrenaicus* have implicitly focused on regions within the species' climatic niche, thereby factoring climate out. If *G. pyrenaicus* requires well-oxygenated waters [26], [28], [29], then steep topography (and hence a high ALT_STD) should be an important predictor. However, *D. moschata* lives well in the slow waters of the lower Ural River basin, perhaps indicating a weaker dependence on well-oxygenated waters, and therefore less importance of steep topography than hitherto proposed also for *G. pyrenaicus* (see [33]). As for HFOOTP, it may not fully represent the type of human impacts that *G. pyrenaicus* is sensitive to, such as the placement of hydroelectric power stations or water sports, as these are not necessarily strongly correlated with the factors that the human footprint is based on, i.e., human population density, land transformation, accessibility and infrastructure [47].

Our results point to dispersal as an additional strong constraint on the distribution of *G. pyrenaicus*, supplemented and probably enhanced by its climate sensitivity. Suitable climatic conditions for *G. pyrenaicus* exist broadly across southern mountainous areas in Europe such as the Alps and in the Balkans (Fig. 5), regions which are currently unoccupied by *G. pyrenaicus* and do not harbour any close relative or likely competitor. The fact that it is absent from these regions in spite of having had at least 15.000 years to disperse to them since the close of the Last Ice Age, provides a strong indication that *G. pyrenaicus* is dispersal limited, probably in large part due to the lack of suitable mountainous habitats between the Pyrenees and the Alps. Presence was also predicted in an area in southern Spain where *G. pyrenaicus* is known to be absent, namely the Sierra Nevada mountains. Its absence here may also be explained by dispersal limitation caused by the wide intervening region of unsuitable conditions or, alternatively, because the area of suitable habitat in the region is too small for the long-term persistence of a *G. pyrenaicus* population (Fig. 3).

### 21^st^ century climate change is a severe threat to *G. pyrenaicus*

All models predicted that the potential distribution of *G. pyrenaicus* would contract under every climate change scenario, although this was especially true in the A1 and A2 scenarios. Every model that included MST predicted the near disappearance of suitable areas for *G. pyrenaicus* from Spain (Fig. 4). The model that included only WBAL predicted less severe but still important reductions in its potential distribution. *In situ* evolutionary adaptation over the next 50–100 years could lessen these predicted negative effects, but is expected to be highly unlikely in reality, as *G. pyrenaicus* has failed to expand into similar warm and dry areas adjacent to its current range during the previous 11,000 years of the present warm period. Anthropogenic habitat fragmentation and population declines would additionally limit its potential for adaptation. Hence, climate change most likely constitutes a major threat to *G. pyrenaicus*, but especially so if the species is directly sensitive to temperature. Studies to more accurately assess the temperature sensitivity of *G. pyrenaicus* will be required in order to measure the severity of the threat that 21^st^ century climate change poses to this species (cf. [13]).

The potentially dramatic range reductions, which may result from climate change over the coming century, combined with the continued fragmentation of suitable habitats, are likely to cause *G. pyrenaicus* to be highly vulnerable to stochastic extinctions [66], as already seen in the Pyrenees [36]. It has been suggested that predation by *Mustela vison* also may constitute an additional threat in the future [33]. Given its broad climatic tolerance in its native North American range, this invasive exotic predator is expected to continue to expand its European range over the next century [67]. However, evidence of the negative impact on populations of *G. pyrenaicus* by this invasive carnivore is still lacking [29]. In all cases, it will be important to focus conservation efforts on improving conditions (notably reducing habitat fragmentation) in the areas that are estimated to be crucial for the long-term survival of *G. pyrenaicus*, i.e., the north-western part of Spain and parts of the Pyrenees.

### Assisted migration as a potential 21^st^ century conservation strategy for *G. pyrenaicus*

**Figure 6:**
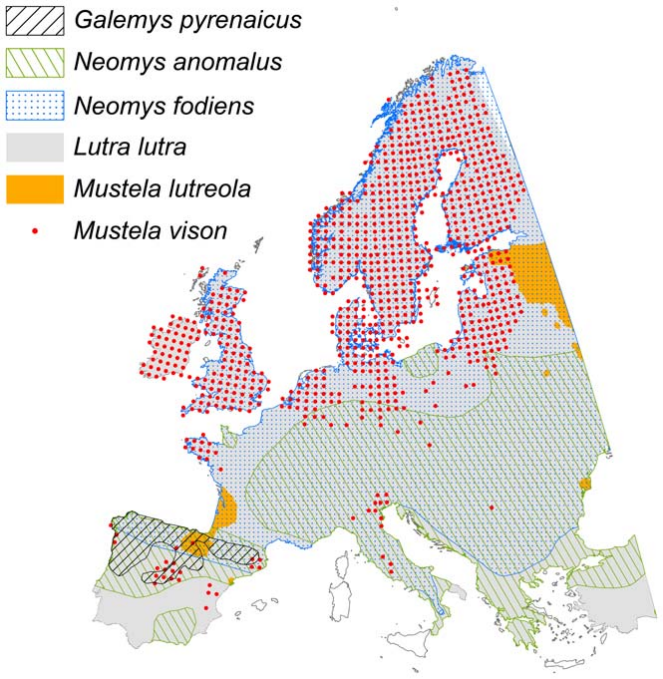
Present distribution of *Galemys pyrenaicus* and its likely competitors and predators in Europe. The range of *Galemys pyrenaicus* currently overlaps with all of its likely competitors and predators in Europe, including those present in the potential introduction areas if assisted migration is implemented [27], [67], [75].

The projections for Europe show large areas with persistently suitable climate for *G. pyrenaicus* beyond its current range; even under the worst future climate scenario, large suitable areas are predicted to occur in Scotland and Scandinavia (Fig. 5). Given the evidence that *G. pyrenaicus* is a poor disperser [36] and is already strongly dispersal-limited on the European scale, having failed to disperse to even relatively nearby suitable areas like the Alps, it is highly unlikely that the species will be able to track the shifting areas of suitable climate on a European scale (cf. [68]). Severe decline or extinction of *G. pyrenaicus* could be prevented if assisted migration beyond its native range is considered an option [24]. Assisted migration is already beginning to be implemented for other species as a management strategy [69] or experimentally [70] and, in the latter case, even using species distribution modeling as guidance, as proposed here. It is, however, a controversial conservation strategy that has led to heated discussions in the scientific literature as well as in the media [69], [71]–[73]. A major concern is the potential for disrupting native biological communities and creating new invasive species problems in the target area [24], [71], [74]. In the case of *G. pyrenaicus*, it is noteworthy that its range already overlaps with its only likely competitors in the potential introduction areas, namely the semi-aquatic shrews *Neomys fodiens* and *N. anomalus* (Fig. 6) [75]. Known predators such as *Lutra lutra*, *Ardea cinerea* and *Mustela vison* in the native range are also currently present in most of the unoccupied suitable areas (Fig. 6). The limited dispersal ability of *G. pyrenaicus* also points to the very low risk that this species will exhibit invasive tendencies at introduction sites. Frameworks as to when to consider assisted migration have been developed and should be used to guide decision making [24], [74], [76]. However, uncertainties and risks associated with assisted migration proposals should always be carefully investigated before implementation of this radical conservation measure. In addition, other conservation strategies in the species' current native range should generally also be considered alongside assisted migration. Improving local conditions, in the case of *G. pyrenaicus* notably by reducing fragmentation due to hydroelectric power stations and contamination of rivers [36] or creating wildlife corridors would probably improve the current conservation status of many of its current populations and increase their robustness to future climatic stress, including at least potentially increasing the possibilities for *in situ* evolutionary adaptation. Nevertheless, as discussed earlier, it seems unrealistic to expect the species to be able to adapt to warmer and drier climate over just 50–100 years, and the results of this study indicate that traditional conservation efforts are unlikely to be enough to ensure the long-term survival of *G. pyrenaicus* in the face of the climatic changes expected for the 21^st^ century [1], [49]. Translocation to higher elevation sites within the current range should also be considered, but the amount of area with suitable temperature will be small (Fig. 4). *Ex situ* captive breeding programmes may offer a short-term solution, but they would need to result in the re-establishment of the species in nature to be effective in the long-term. Hence, assisted migration may well become a necessary future conservation strategy for *G. pyrenaicus*. Nonetheless, if assisted migration is to be considered for practical implementation, field trials should be performed to test for any unwanted side effects of introductions to a given area and to assess its general likelihood of success [74].

### Conclusions

The current climate, in particular water balance and mean summer temperature, appears to be the main determinant of the present distribution of *G. pyrenaicus*, even though dispersal probably also strongly limits the distribution at a broader scale. This restricted mountain endemic is therefore likely to be highly sensitive to global warming over the next century; a very strong negative impact is expected even for the less severe climate change scenarios. Future suitable areas for *G. pyrenaicus* may exist in other parts of Europe far beyond its current range. Given the clearly limited dispersal abilities of *G. pyrenaicus,* assisted migration is therefore potentially an essential component of the climate-change-integrated conservation strategy for the species. Future studies on *G. pyrenaicus* should concentrate on clarifying its temperature sensitivity, as the severity of the global warming threat strongly depends on its sensitivity to high temperatures *per se*. The results of the present study confirm the conclusion of Ohlemüller et al. [23] that many endemic species may be highly vulnerable to a warming climate.

## Supporting Information

Figure S1 Agreement between modeled and observed distributions of *Galemys pyrenaicus*. Assessment of the agreement between modeled and observed distributions according to Cohen's kappa statistic for the three suitability ranges of BIOCLIM (BIO) models (i.e., minimum and maximum, 2.5^th^ and 97.5^th^ percentiles and 10^th^ and 90^th^ percentiles of the observed environmental values within the current range in the study area) and the MAXENT models. The included predictor variables are: Model 2: ALT_STD, HFOOTP, MST, MWT and WBAL; Model 4: MST and WBAL; Model 5: ALT_STD, MST and WBAL; Model 6: WBAL; Model 7: MST.
(0.08 MB TIF)

Figure S2 Potential present and future distribution in Spain according to BIOCLIM. BIOCLIM model predictions of the present and future potential distribution of *Galemys pyrenaicus* in Spain at a 10×10 km resolution based on (A) WBAL and (B) MST. Maximum and minimum, 2.5^th^ and 97.5^th^ percentiles and 10^th^ and 90^th^ percentiles of the variables are shown. (C) Ensemble prediction: Agreement on the predicted distribution based on the 2.5^th^ and 97.5^th^ percentiles of the variables among all five final MAXENT models. The colours indicate the number of models predicting presence for each grid cell ranging from 0 to 5.
(1.64 MB TIF)

## References

[1] Meehl GA, Stocker TF, Collins WD, Friedlingsteing P, Gaye AT, Solomon S, Qin D, Manning M, Chen Z, Marquis M (2007). Global Climate Projections.. Climate Change 2007: The Physical Science Basis. Contribution of Working Group I to the Fourth Assessment Report of the Intergovernmental Panel on Climate Change.

[2] Walther GR, Post E, Convey P, Menzel A, Parmesan C (2002). Ecological responses to recent climate change.. Nature.

[3] Root TL, Price JT, Hall KR, Schneider SH, Rosenzweig C (2003). Fingerprints of global warming on wild animals and plants.. Nature.

[4] Alcamo J, Moreno JM, Nováky B, Bindi M, Corobov R, Parry ML, Canziani OF, Palutikof JP, van der Linden PJ, Hanson CE (2007). Europe.. Climate Change 2007: Impacts, Adaptation and Vulnerability. Contribution of Working Group II to the Fourth Assessment Report of the Intergovernmental Panel on Climate Change.

[5] Fischlin A, Midgley GF, Price JT, Leemans R, Gopal B, Parry ML, Canziani OF, Palutikof JP, van der Linden PJ, Hanson CE (2007). Ecosystems, their properties, goods, and services.. Climate Change 2007: Impacts, Adaptation and Vulnerability. Contribution of Working Group II to the Fourth Assessment Report of the Intergovernmental Panel on Climate Change.

[6] Moritz C, Patton JL, Conroy CJ, Parra JL, White GC (2008). Impact of a century of climate change on small-mammal communities in Yosemite National Park, USA.. Science.

[7] Rosenzweig C, Karoly D, Vicarelli M, Neofotis P, Wu Q (2008). Attributing physical and biological impacts to anthropogenic climate change.. Nature.

[8] Aublet J, Festa-Bianchet M, Bergero D, Bassano B (2009). Temperature constraints on foraging behaviour of male Alpine ibex (*Capra ibex*) in summer.. Oecologia.

[9] Grayson DK (2000). Mammalian responses to middle Holocene climatic change in the Great Basin of the Western United States.. J Biogeogr.

[10] Svenning JC (2003). Deterministic Plio-Pleistocene extinctions in the European cool-temperate tree flora.. Ecol Lett.

[11] Grayson DK, Delpech F (2005). Pleistocene reindeer and global warming.. Conserv Biol.

[12] Sala OE, Stuart Chapin F, Armesto JJ, Berlow E, Bloomfield J (2000). Global biodiversity scenarios for the year 2100.. Science.

[13] Williams SE, Bolitho EE, Fox S (2003). Climate change in Australian tropical rainforests: an impending environmental catastrophe.. P Roy Soc Lond B Bio.

[14] Thomas CD, Cameron A, Green RE, Bakkenes M, Beaumont LJ (2004). Extinction risk from climate change.. Nature.

[15] Thuiller W, Lavorel S, Araújo MB, Sykes MT, Prentice IC (2005). Climate change threats to plant diversity in Europe.. P Natl Acad Sci USA.

[16] Levinsky I, Skov F, Svenning JC, Rahbeck C (2007). Potential impacts of climate change on the distributions and diversity patterns of European mammals.. Biodivers Conserv.

[17] Welbergen JA, Klose SM, Markus N, Eby P (2008). Climate change and the effects of temperature extremes on Australian flying-foxes.. P Roy Soc Lond B Bio.

[18] Gaston JK (1994). Rarity..

[19] Svenning JC, Skov F (2004). Limited filling of the potential range in European tree species.. Ecol Lett.

[20] Araújo MB, Nogués-Bravo D, Diniz-Filho JAF, Haywood AM, Valdes PJ (2008). Quaternary climate changes explain diversity among reptiles and amphibians.. Ecography.

[21] Jetz W, Rahbek C (2002). Geographic range size and determinants of avian species richness.. Science.

[22] Jansson R (2003). Global patterns in endemism explained by past climatic change.. P Roy Soc Lond B Bio.

[23] Ohlemüller R, Anderson BJ, Araújo MB, Butchart SHM, Kudma O (2008). The coincidence of climatic and species rarity: high risk to small-range species from climate change.. Biol Lett.

[24] Hoegh-Guldberg O, Hughes L, McIntyre S, Lindendmayer DB, Parmesan C (2008). Assisted colonization and rapid climate change.. Science.

[25] Svenning JC, Fløjgaard C, Morueta-Holme N, Lenoir J, Normand S (2009). Big moving day for biodiversity? A macroecological assessment of the scope for assisted colonization as a conservation strategy under global warming.. IOP Conf Ser: Earth Environ Sci.

[26] Palmeirim JM, Hoffmann RS (1983). *Galemys pyrenaicus*.. Mamm Species.

[27] IUCN (2007). European Mammal Assessment.. http://ec.europa.eu/environment/nature/conservation/species/ema.

[28] Nores C, Palacios B, Martín JA, Vázquez I, González J (1999). Informe sobre la situación del Desmán Ibérico (*Galemys pyrenaicus*) en España..

[29] Palomo LJ, Gisbert J, Blanco JC (2007). Atlas y Libro Rojo de los Mamíferos Terrestres de España..

[30] Schreuder A (1940). A revision of the fossil water-moles (Desmaninae).. Arch Néerl Zool.

[31] Degerbøl M (1964). Some remarks on Late- and Post-Glacial vertebrate fauna and its ecological relations in Northern Europe.. J Anim Ecol.

[32] Smirnov NG, Ponomarev DV (2007). News about the past distribution of the Desman (*Desmana moschata* L.).. Dokl Biol Sciences.

[33] Nores C, Ojeda F, Ruano A, Villate I, González J (1992). Aproximación a la metodología y estudio del area de distribución, estatus de población y selección de hábitat del desmán (*Galemys pyrenaicus*) en la Península Ibérica..

[34] Castel E (2003). L'almesquera, un mamífer enigmàtic.. Hàbitats.

[35] Gisbert J, García-Perea R (2003). Catálogo Nacional de Especies Amenazadas, *Galemys pyrenaicus*..

[36] Aymerich P (2004). Els micromamífers semiaquàtics d'Andorra: distribució i estat de conservació.. Hàbitats.

[37] Cabria MT, Rubines J, Gómez-Moliner B, Zardoya R (2006). On the phylogenetic position of a rare Iberian endemic mammal, the Pyrenean desman (*Galemys pyrenaicus*).. Gene.

[38] Guisan A, Thuiller W (2005). Predicting species distribution: offering more than simple habitat models.. Ecol Lett.

[39] Pearson RG, Raxworthy CJ, Nakamura M, Peterson AT (2007). Predicting species distributions from small numbers of occurrence records: a test case using cryptic geckos in Madagascar.. J Biogeogr.

[40] Dormann CF (2007). Promising the future? Global change projections of species distributions.. Basic Appl Ecol.

[41] Botkin DB, Saxe H, Araújo MB, Betts R, Bradshaw RHW (2007). Forecasting the effects of global warming on biodiversity.. Bioscience.

[42] Svenning JC, Skov F (2007). Ice age legacies in the geographical distribution of tree species richness in Europe.. Global Ecol Biogeogr.

[43] Aymerich P, Gosàlbez J (2004). La prospección de excremetos como metodología para el estudio de la distribución de los musgaños..

[44] Pearce JL, Boyce MS (2006). Modelling distribution and abundance with presence-only data.. J Appl Ecol.

[45] Hijmans RJ, Cameron SE, Parra JL, Jones PG, Jarvis A (2005). Very high resolution interpolated climate surfaces for global land areas.. Int J Climatol.

[46] CIESIN (Center for International Earth Science Information Network), CIAT (Columbia University and Centro Internacional de Agricultura Tropical) (2005). Population Density Grids.. http://sedac.ciesin.columbia.edu/gpw.

[47] Sanderson EW, Jaiteh M, Levy MA, Redford KH, Wannebo AV (2002). The human footprint and the last of the wild.. Bioscience.

[48] Buermann W, Saatchi S, Smith TB, Zutta BR, Chaves JA (2008). Predicting species distributions across the Amazonian and Andean regions using remote sensing data.. J Biogeogr.

[49] Mitchell TD, Carter TR, Jones PD, Hulme M, New M (2004). A comprehensive set of high-resolution grids of monthly climate for Europe and the globe: the observed record (1901–2000) and 16 scenarios (2001–2100).. Tyndall Centre, Norwich: Tyndal Working Paper.

[50] Phillips SJ, Anderson RP, Schapire RE (2006). Maximum entropy modeling of species geographic distributions.. Ecol Model.

[51] Elith J, Graham H, Anderson P, Dudik M, Ferrier S (2006). Novel methods improve prediction of species' distributions from occurrence data.. Ecography.

[52] Phillips SJ, Dudik M (2008). Modeling of species distributions with Maxent: new extensions and a comprehensive evaluation.. Ecography.

[53] Araújo MB, New M (2007). Ensemble forecasting of species distributions.. Trends Ecol Evol.

[54] Pearson RG, Thuiller W, Araújo MB, Martinez-Meyer E, Brotons L (2006). Model-based uncertainty in species range prediction.. J Biogeogr.

[55] Nix HA, Longmore R (1986). A biogeographic analysis of Australian elapid snakes.. Atlas of elapid snakes of Australia.

[56] Hernandez PA, Graham CH, Master LL, Albert DL (2006). The effect of sample size and species characteristics on performance of different species distribution modeling methods.. Ecography.

[57] Fielding AH, Bell JF (1997). A review of methods for the assessment of prediction errors in conservation presence/absence models.. Environ Conserv.

[58] Thuiller W, Brotons L, Araújo MB, Lavorel S (2004). Effects of restricting environmental range of data to project current and future species distributions.. Ecography.

[59] Ficetola GF, Thuiller W, Miaud C (2007). Prediction and validation of the potential global distribution of a problematic alien invasive species - the American bullfrog.. Divers Distrib.

[60] Pearman PB, Randin CF, Broennimann O, Vittoz P, van der Knaap WO (2008). Prediction of plant species distributions across six millennia.. Ecol Lett.

[61] Fløjgaard C, Normand S, Skov F, Svenning JC (2009). Ice age distributions of European small mammals: insights from species distribution modelling.. J Biogeogr.

[62] Peterson AT, Papes M, Eaton M (2007). Transferability and model evaluation in ecological niche modeling: a comparison of GARP and Maxent.. Ecography.

[63] Tinoco BA, Astudillo PX, Latta SC, Graham CH (2009). Distribution, ecology and conservation of an endangered Andean hummingbird: the Violet-throated Metaltail (*Metallura baroni*).. Bird Conserv Int.

[64] Nogués-Bravo D, Rodríguez J, Hortal J, Batra P, Araújo MB (2008). Climate change, humans, and the extinction of the woolly mammoth.. PLoS Biol.

[65] Pearson RG, Dawson TP (2003). Predicting the impacts of climate change on the distribution of species: are bioclimate envelope models useful?. Global Ecol Biogeogr.

[66] Mira A, Marques CC, Santos SM, Rosário IT, Mathias ML (2008). Environmental determinants of the distribution of the Cabrera vole (*Microtus cabrerae*) in Portugal: Implications for conservation.. Z Saugetierkd.

[67] Mitchell-Jones AJ, Amori G, Bogdanowicz W, Krystufek B, Reijnders PJH (1999). The atlas of European mammals..

[68] Skov F, Svenning JC (2004). Potential impact of climatic change on the distribution of forest herbs in Europe.. Ecography.

[69] Marris E (2008). Moving on assisted migration.. Nature Reports: Climate Change.

[70] Willis SG, Hill JK, Thomas CD, Roy DB, Fox R (2009). Assisted colonization in a changing climate: a test-study using two U.K. butterflies.. Conserv Lett.

[71] Ricciardi A, Simberloff D (2009). Assisted colonization is not a viable conservation strategy.. Trends Ecol Evol.

[72] Vitt P, Havens K, Hoegh-Guldberg O (2009). Assisted migration: part of an integrated conservation strategy.. Trends Ecol Evol.

[73] Seddon PJ, Armstrong DP, Soorae P, Launay F, Walker S (2009). The risks of assisted colonization.. Cons Biol.

[74] McLachlan JS, Hellmann JJ, Schwartz MW (2007). A framework for debate of assisted migration in an era of climate change.. Conserv Biol.

[75] IUCN (2009). IUCN Red List of Threatened Species.. http://iucnredlist.org.

[76] Richardson DM, Hellmann JJ, McLachlan JS, Sax DF, Schwartz MW (2009). Multidimensional evaluation of managed relocation.. P Natl Acad Sci.

[77] Prentice IC, Cramer W, Harrison SP, Leemans R, Monserud RA (1992). A global biome model based on plant physiology and dominance, soil properties and climate.. J Biogeogr.

