# Supplementary figures and images for: Climate Change Risks and Conservation Implications for a Threatened Small-Range Mammal Species
Source: PLoS One. 2010 Apr 29;5(4):e10360. doi: 10.1371/journal.pone.0010360 (PMC2861593; doi:10.1371/journal.pone.0010360)

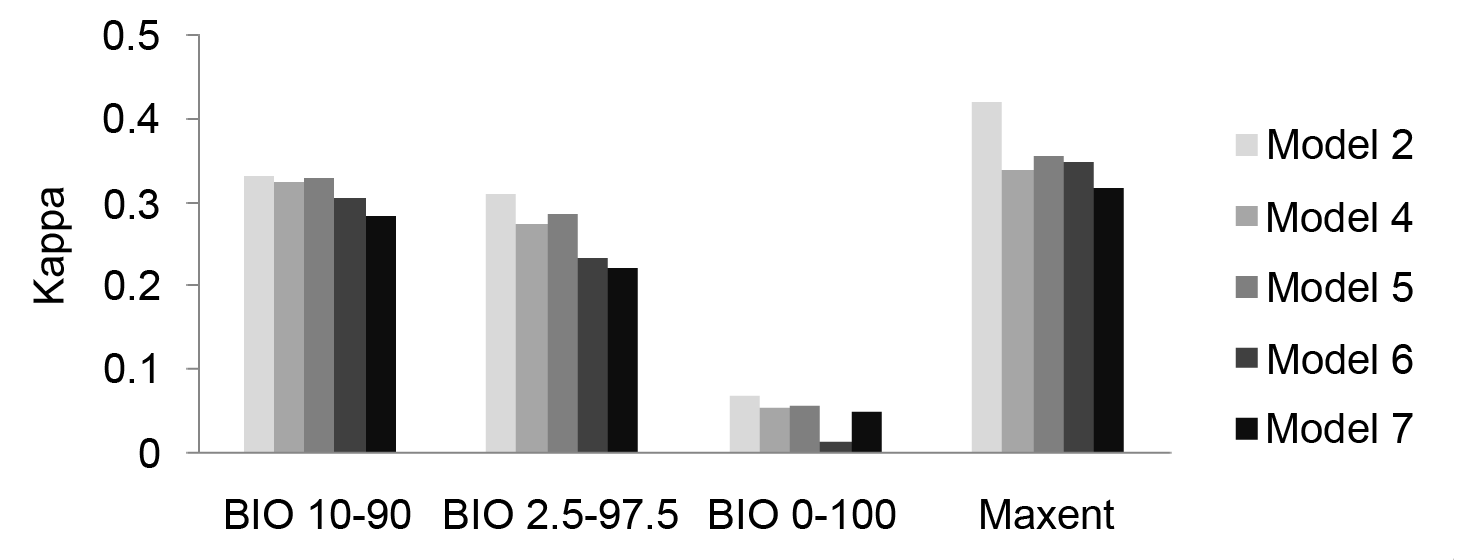

Supplement: Figure S1 — Agreement between modeled and observed distributions of Galemys pyrenaicus. Assessment of the agreement between modeled and observed distributions according to Cohen's kappa statistic for the three suitability ranges of BIOCLIM (BIO) models (i.e., minimum and maximum, 2.5th and 97.5th percentiles and 10th and 90th percentiles of the observed environmental values within the current range in the study area) and the MAXENT models. The included predictor variables are: Model 2: ALT_STD, HFOOTP, MST, MWT and WBAL; Model 4: MST and WBAL; Model 5: ALT_STD, MST and WBAL; Model 6: WBAL; Model 7: MST. (0.08 MB TIF) [file pone.0010360.s001.tif]

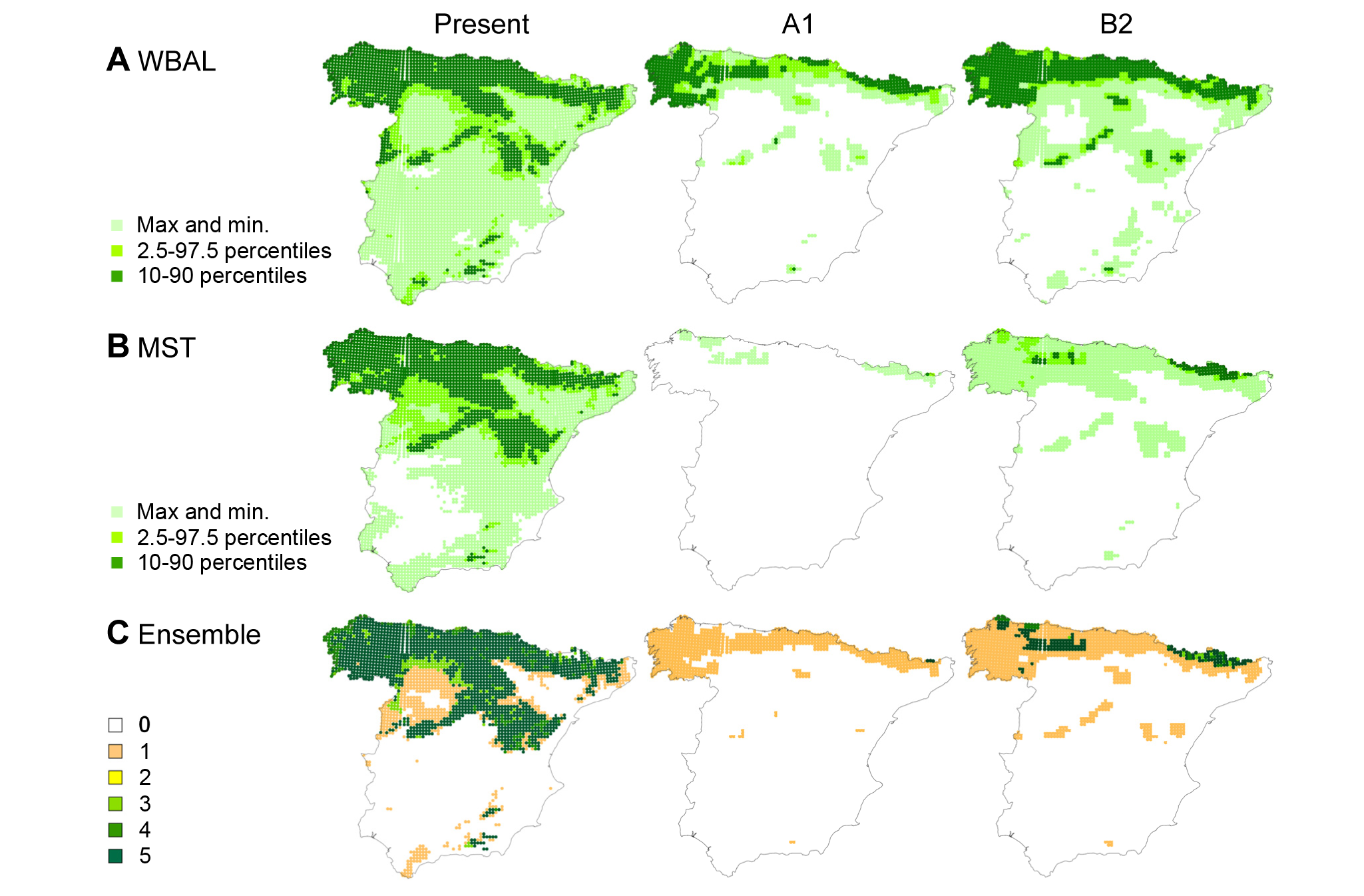

Supplement: Figure S2 — Potential present and future distribution in Spain according to BIOCLIM. BIOCLIM model predictions of the present and future potential distribution of Galemys pyrenaicus in Spain at a 10×10 km resolution based on (A) WBAL and (B) MST. Maximum and minimum, 2.5th and 97.5th percentiles and 10th and 90th percentiles of the variables are shown. (C) Ensemble prediction: Agreement on the predicted distribution based on the 2.5th and 97.5th percentiles of the variables among all five final MAXENT models. The colours indicate the number of models predicting presence for each grid cell ranging from 0 to 5. (1.64 MB TIF) [file pone.0010360.s002.tif]
